# Potential Risk of Malposition of Nasogastric Tube Using Nose-Ear-Xiphoid Measurement

**DOI:** 10.1371/journal.pone.0088046

**Published:** 2014-02-10

**Authors:** Yen-Chun Chen, Lien-Yen Wang, Yu-Jun Chang, Chao-Pin Yang, Tsung-Ju Wu, Fung-Ru Lin, Sen-Yung Liu, Ta-Sen Wei

**Affiliations:** 1 Department of Physical Medicine and Rehabilitation, Changhua Christian Hospital, Changhua, Taiwan; 2 Department of Nuclear Medicine, Changhua Christian Hospital, Changhua, Taiwan; 3 Laboratory of Epidemiology and Biostatistics, Changhua Christian Hospital, Changhua, Taiwan; 4 School of Medicine, Chung Shan Medical University, Taichung, Taiwan; The Ohio State University, United States of America

## Abstract

**Background:**

Correct placement of nasogastric tubes provide proper functionality and maximize benefit and minimize risk. The Nose-Ear-Xiphoid (NEX) body surface estimate method is a long-lasting technique, and this study was conducted to evaluate the correlation between NEX method and the secure insertion depth of nasogastric tube.

**Materials and Methods:**

Thirty patients with nasogastric tube insertion who received whole body positron emission tomography with computerized tomography scan (PET-CT) were recruited. All data were gathered in the image center, which included Nose-Ear (NE), Ear-Xiphoid (EX), Nose-Ear-Xiphoid (NEX), glabella-xiphoid (GX) and glabella-umbilicus (GU) lengths. The distances of the inserted portion of the nasogastric tube between the cardiac and the nostril were measured by multiplanar reconstruction algorithm.

**Results:**

Only one patient successfully placed all side-holes into the stomach while using NEX method to estimate inserting depth. Twenty-nine patients (96.7%) failed to place correctly. Fourteen participants had one or more side-holes in both the esophagus and the stomach sides. Fifteen patients could not pass through any side-hole across the gastroesophageal junction. They had shorter EX distances (p = 0.02), but no difference among the NE distances. Body height had the highest statistical correlation with nasogastric tube length (adjusted R^2^ = 0.459), as compared with the NEX, GX and GU body surface methods.

**Conclusion:**

This study suggests that NEX method is inappropriate for adult patients to estimate the ideal inserting length of nasogastric tube. Physicians should realize these underinsertions with any side-hole above the gastroesophageal junctions may increase the potential risk of complications.

## Introduction

Since the momentous invention of the nasogastric tube (NG tube) by Dr. Abraham Louis Levin in 1921, it has become one of the most practical and even effective managements in modern medical history. [1] Besides establishing a safe pathway into the stomach without misdirection in patients with dysphagia or uncooperative behavior, NG intubation also provides symptomatic relief and decompression while obstruction of the small bowel or gastric outlet, ileus and severe pancreatitis. [2]–[3] It will also allow for gastric lavage in the management of patients with hematemesis, hematochezia, drug overdose and poison intake. [4]

NG tube insertion is not a benign procedure, and the accurate placements of these tubes provide the proper functionality and maximize benefits and minimize risks. [5] Otherwise, complications have been described in the related medical literatures such as aspiration, pneumothorax, transbronchial intubation of the right pleural space, esophageal perforation and tears, and even intracranial placement. [6]–[8] Verification of NG tube placement is recommended after initial tube insertion and before each intermittent feeding or medication administration. [9]

The correct placement depends on the proper depth of inserted tube and appropriate location of catheter-tip. Previous studies focused on the confirmation tests of proper placement. Auscultating borborygmus over the epigastrium during air injection and the use of PH test paper has been reported. [12] Besides, there are some ways to estimate the proper depth of the NG tube before the insertion technique. Body surface measurement of the distance from the nostril to the lobule of auricle and then to the xiphoid process, referred to as Nose-Ear-Xiphoid (NEX) method, is the most common and long-lasting technique and has even been published as the gold standard of NG tube insertion. [5], [10] However, there are still many aspirations of gastric content that predispose tube-dependent patients to pneumonia while in clinical practice, especially those who are critically ill and have consciousness disturbance. [11] Few studied discussed the safety of NEX body surface measurement even after all these years.

On the insertion portion of the NG tube, there are about 3 or 4 side-holes which allow the instilled liquid to drain away. The distances between the proximal hole and the catheter-tip vary due to the different types or brands of tubes, and it is easy to neglect its clinical importance during intubation. Physicians should confirm the proper placement of tubes into the stomach especially all the distal parts of the tube with side-holes. If there are some proximal side-holes still in the esophagus or higher than the gastroesophageal junction, the patients might be prone to have the risk of aspiration. [13]

In order to investigate the biological significance of secure insertion depth, the aim of this retrospective-designed study was to evaluate the correlation between the NEX body surface method and secure insertion depth of NG tube.

## Methods

### Ethics Statement

This retrospective study design was approved by the Institutional Review Board of the Changhua Christian Hospital. The informed consent was specifically waived by the approving ethics committee. The waiver of consent did not adversely affect the rights and welfare of the subjects. All research in current study has been conducted under the surveillance of Institutional Review Board.

### Participants

The study was conducted from March 2011 to September 2012 at Changhua Christian Hospital, Chunghua, Taiwan. Adult patients (≥18 years old) with NG tube insertion who received whole body positron emission tomography with non-contrast and low-dose computerized tomography scan (PET-CT) (GEMINI GXL 16 PET/CT system, PHILIPIS, America) were retrospectively enrolled into the study. Patients were excluded if they had prior esophageal surgery or known congenital abnormalities of the esophagus.

### Data Collect

**Figure 1:**
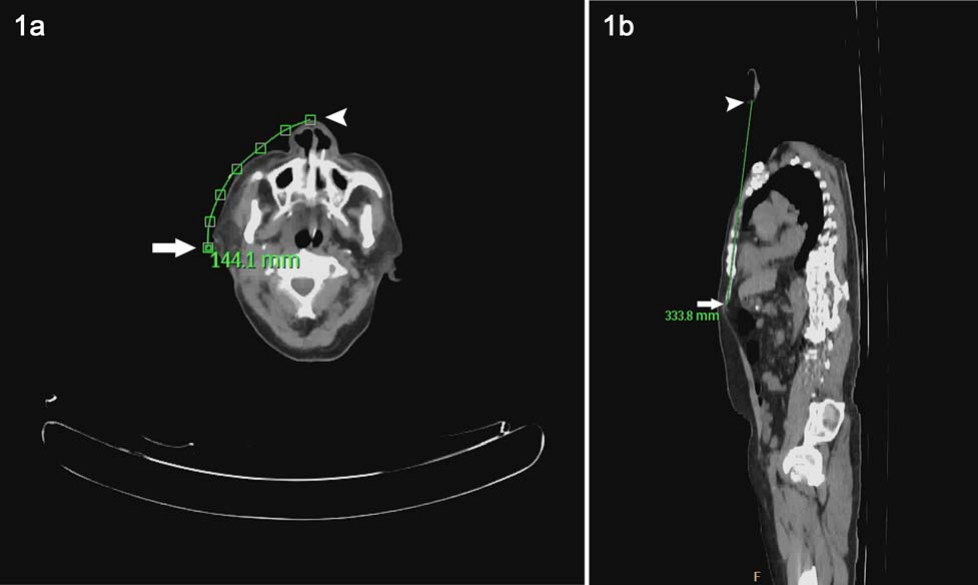
a. The curved length of Nose-Ear (NE) was measured from the nostril (Arrow) to the lobule of auricle (Arrowhead) by tracing five more points along the face surface between the two ends. b. The linear Ear-Xiphoid (EX) length is measured from the ear lobe (Arrow) to the lower tip of xiphoid (Arrowhead).

The image data was gathered and analyzed in the PET scan center. We used the Define Curve function of the CT viewer planar mode of PHILIPS GEMINI GLX EBW V3.5.2.2260 to perform the desired path length measurement. The distance from the nostril to the lobule of auricle and then to the xiphoid process was collected, known as the NEX measurement. The curved length of Nose-Ear (NE) was measured from the nostril and on five more points along the face surface between the nostril and the lobule of auricle. (Fig 1a) Ear-Xiphoid (EX) length is linear and measured from ear lobe to the lower tip of xiphoid. (Fig 1b)

Previous literature reported some other body surface methods, for example, the glabella-xiphoid (GX) and glabella-umbilicus (GU) measurements. [14] These two linear distances were also tested, and GX length was measured from the glabella (the space between the eyebrows and above the nose) to the xiphoid process. GU length was measured from the glabella to the umbilicus.

**Figure 2:**
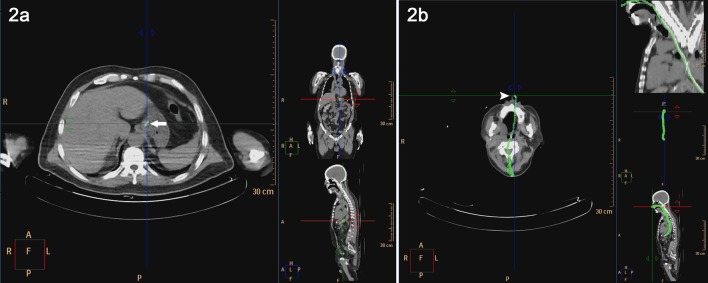
a. Pointed out the NG tube (Arrow) at the transverse level of the cardia. b. Every locations of NG tube are found on the transaxial images (5 mm thickness) with tracing upward till the nostril (Arrowhead). Curved multiplanar algorithm which represents the NG tube is reconstructed and measured.

For the measurement of inserted portion of NG tube between the antrum cardiacum and the nostril, we pointed out the location of NG tube at the transverse level of the cardia. (Fig 2a) Every position of NG tube on the transaxial CT images (5 mm thickness) was found, and then we traced the NG tube upwards until reaching the nostril area. Finally, the curved multiplanar reconstruction algorithm of the CT viewer showed the traced path of NG tube on a coronal CT image in 2-D mode. (Fig 2b) The length of inserted portion of NG tube was obtained by measuring the curved path along traced points.

**Figure 3:**
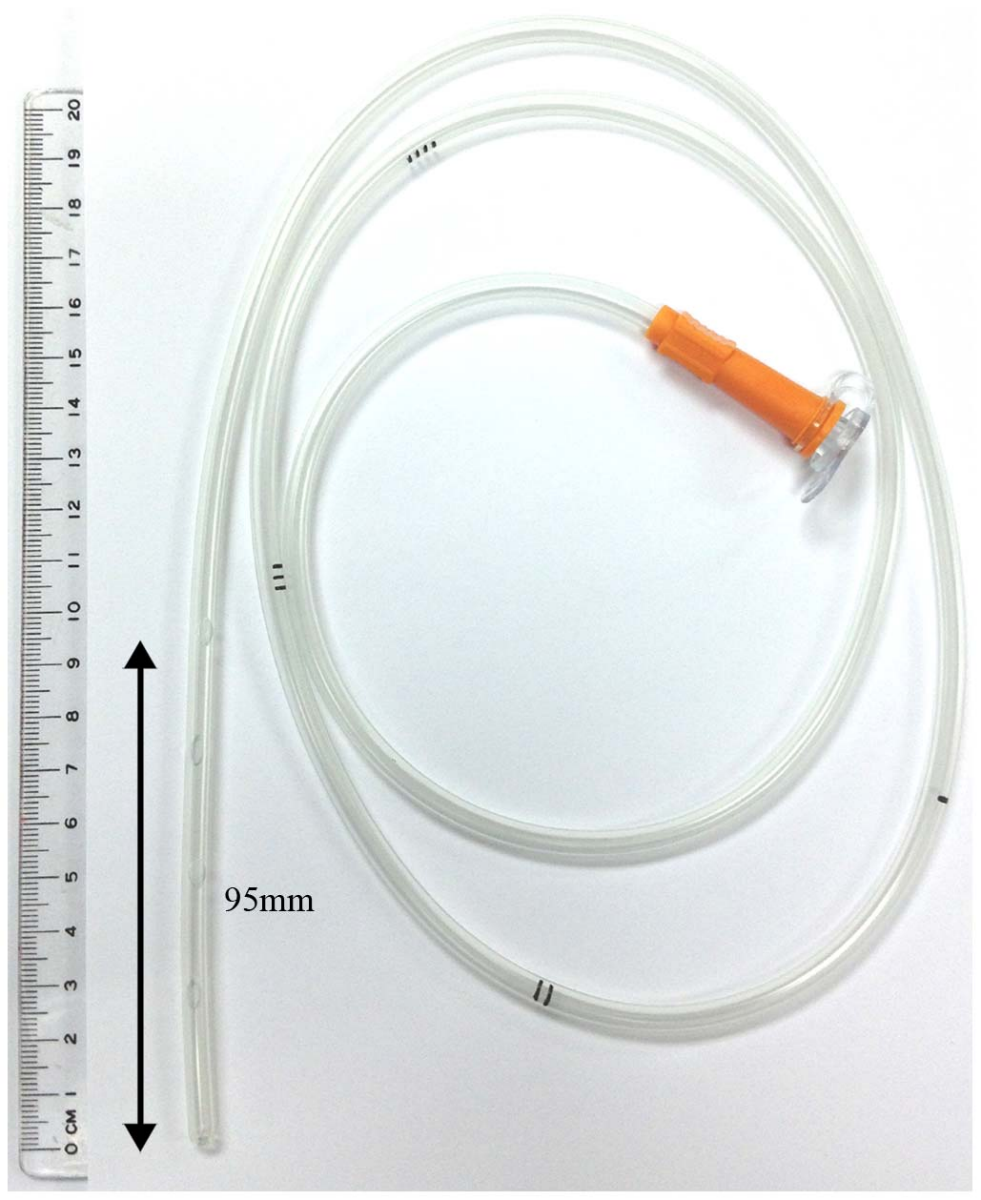
A common type of NG tube is 125 cm in length and with marks at 45, 55, 65 and 75 cm. Four side-holes are located at the insertion end, and the distances to the catheter-tip are 95, 73, 51, and 28 mm respectively.

The distances between the proximal side-hole and the catheter-tip varied among the commercially available NG tubes in the market. In one of the most common types of tube, there are four side-holes on the surface of distal catheter and length from the proximal side-hole to catheter-tip is 95 mm. (polyvinylchloride; Symphon Chemical Corp, New Taipei City, Taiwan) (Fig 3) Distances between the proximal second, third, and forth side-hole to the tube-end are 73, 51, and 28 mm, respectively.

Take side-holes distances into consideration, the inserting depth should add additional 95 mm to make sure the secure placement that all four side-holes are inserted passing through the esophagogastric junction. Therefore, the tube content can outflow into stomach and avoid residual in the esophagus. While using the NEX estimate method, patients usually had insufficient NEX lengths. Additional lengths to secure side-hole placement were calculated for each of participants.

### Statistical Analysis

All continuous variables are presented as mean and standard deviation (SD). The descriptive statistics also included median and extreme values. Comparisons between patient groups were performed by using the Mann-Whitney U Test for continuous data. Distribution and independence of variables were checked by Shapiro-Wilk test. All dataset follows a normal distribution. Linear regression models were performed to identify the correlations between the NG length from cardia to nostril and the predict models of patient height, and three body surface methods (NEX, GX, and GU lengths). Correlations between the additional lengths to secure side-holes placement and the NEX lengths/heights were analyzed by linear regression models. The level of statistical significance was set up P less than .05. The statistical software SPSS (version 15.0; SPSS Inc.) was used for all statistical analyses.

## Results

A total of 31 patients were screened between March 2011 and September 2012. One patient was excluded for previous esophagus surgery. The remaining 30 patients were enrolled into the study (27 men, 3 women; mean age, 57.7±10.4y). Most of our patients had the diagnosis of head and neck, or lung cancer and received whole body PET-CT. The mean height of patients was 162.7±7.4 cm; the mean length of NG tube from nostril to the cardia of stomach was 473.5±27.9 mm. (Table 1) The mean distance from the nostril to the lobule of auricle and then to the xiphoid process (NEX length) was 511.5±29.9 mm. The mean GX and GU lengths were 403.3±26.5 and 559.0±34.6 mm, respectively.

**Table 1:** Descriptive statistics and comparison between the “No side-hole pass” and the opposite group.

	Total (n = 30)			No side-hole pass (n = 15)			At least one side-hole pass (n = 15)			
	Mean	SD	Median (Min-Max)	Mean	SD	Median (Min-Max)	Mean	SD	Median (Min-Max)	P-value
Age	57.7	10.4	56.0 (33.0–75.0)	57.8	9.4	51.0 (39.0–71.0)	57.7	11.7	56.0 (33.0–75.0)	0.950
Height (cm)	162.7	7.4	164.3 (148.0–177.0)	164.1	6.1	157.0 (155.0–177.0)	161.3	8.5	161.0 (148.0–174.0)	0.339
NG length cardia to nostril (mm)	473.5	27.9	474.6 (422.2–522.1)	488.6	23.4	464.7 (448.1–522.1)	458.4	23.9	454.9 (422.2–496.1)	0.003
NE length (mm)	153.0	10.0	152.2 (127.6–171.2)	151.9	11.0	145.9 (127.6–171.2)	154.0	9.2	150.2 (139.8–169.5)	0.772
EX length (mm)	358.5	25.6	356.3 (301.6–424.1)	348.1	22.8	339.2 (301.6–380.7)	368.9	24.5	371.7 (315.5–424.1)	0.020
NEX length (mm)	511.5	29.9	509.5 (453.1–593.6)	500.0	27.0	483.7 (453.1–540.3)	523.0	28.9	518.6 (471.2–593.6)	0.036
GX length (mm)	403.3	26.5	402.2 (339.6–447.6)	401.8	26.7	390.1 (339.6–436.4)	404.8	27.1	407.5 (357.8–447.6)	0.787
GU length (mm)	559.0	34.6	565.6 (479.6–607.5)	557.6	31.2	545.3 (479.6–604.1)	560.3	38.8	579.1 (483.3–607.5)	0.494

P-value by Mann-Whitney U Test.

NG: nasogastric; NE: Nose-Ear; EX: Ear-Xiphoid; NEX: Nose-Ear-Xiphoid; GX: Glabella-Xiphoid; GU: Glabella-Umbilicus; SD: standard deviation; Min: minimum; Max: maximum.

Among 30 patients, only 1 patient (3.3%) had a long enough NEX distance to pass all four side-holes into the stomach while using NEX method to estimate the tube depth. Twenty-nine people (96.7%) had an inappropriate side-hole position higher than the esophagogastric junction. Three holes passing through with one residual hole outside the confines of the stomach were noted in 2 people. Eight patients had 2 holes in the stomach and the remaining other 2 holes in the esophagus. Four patients experienced one side-hole passing through and 3 residual holes in the esophagus. There were 15 remaining patients (50%) that had inappropriate estimated insertion depth and could not even pass any side-holes through into the stomach.

In conclusion, fifteen people failed to pass through any side-holes, and 14 had one or more side-holes in both the esophagus and the stomach. Only one patient had correct side-hole placement into the stomach. Comparing the 15 patients who failed to pass through any side-holes with the opposite 15 patients, they had significantly shorter EX distances (p = 0.02) while no difference among the NE distances was noted (p = 0.77). (Table 1)

By using the GX measure to estimate NG tube length, no person had an enough long GX length to pass through side-holes. Fourteen (45.2%) patients had long enough predicted distances to pass all four side-holes into the stomach while using the GU method to estimate tube depth.

In linear regression models between the NG length and variables, body height is the most valuable predictor which the correlation coefficient is 0.691 and the adjusted R^2^ is 0.459 (p<0.001). (Table 2) With the predictor of NEX method, the correlation coefficient is 0.429 and the adjusted R^2^ is 0.155 (p = 0.018). With the variables of GX and GU lengths, the correlation coefficients are 0.570 and 0.355, respectively.

**Table 2:** Linear Regression Models on the NG length.

Model	Predictors	β	SE	Std β	95% C.I. for β			P-value		Adj R^2^		
1	(Constant)	145.879	83.559		−25.283	-	317.041	0.092		0.459		
	Height (cm)	2.597	0.513	0.691	1.546	-	3.648	<0.001				
2	(Constant)	363.611	81.627		196.405	-	530.816	<0.001		0.155		
	NEX length (mm)	0.401	0.159	0.429	0.074	-	0.727		0.018			
3	(Constant)	326.933	66.006		191.726	-	462.140		<0.001		0.300	
	GX length (mm)	0.599	0.163	0.570	0.264	-	0.933		0.001			
4	(Constant)	409.114	79.583		246.095	-	572.132		<0.001		0.094	
	GU length (mm)	0.285	0.142	0.355	−0.006	-	0.576		0.055			

NG: nasogastric; NEX: Nose-Ear-Xiphoid; GX: Glabella-Xiphoid; GU: Glabella-Umbilicus; β: regression coefficient; Std β: standardized regression coefficient; Adj R^2^: adjusted R^2^.

**Figure 4:**
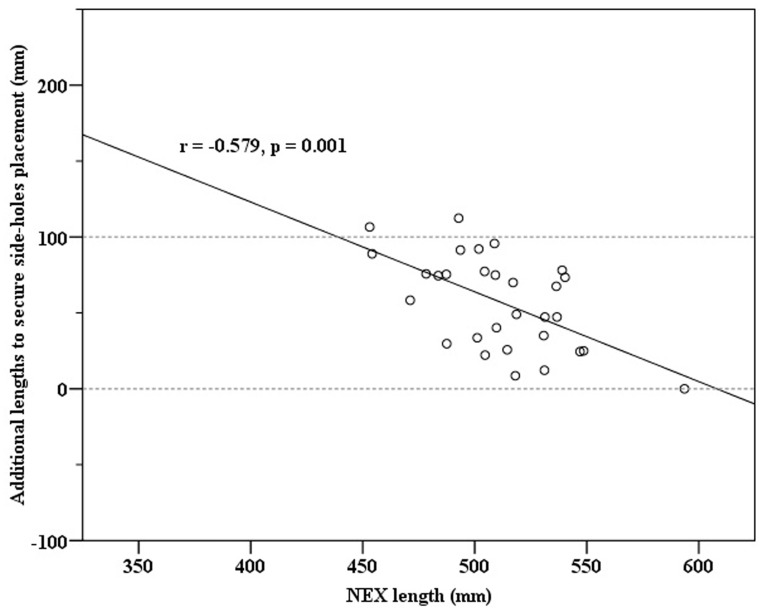
Scatter plot shows the significant negative correlation between NEX length and additional length to secure side-hole placement.

By using linear regression model on the additional length to secure side-hole placement, predictor of NEX length showed that the correlation coefficient is −0.579 and the adjusted R^2^ is 0.312 (p = 0.001). (Table 3) The distribution of these two variables was statistically meaningful and revealed strong negative correlations. (Figure 4) However, height did not show a significant association with regards to the additional length to secure side-holes placement. (p = 0.829)

**Table 3:** Linear regression models on the additional length to secure side-hole placement.

Model	Predictors	β	SE	Std β	95% C.I. for β			P-value		Adj R^2^		
1	(Constant)	363.611	81.627		196.405	-	530.816	<0.001		0.312		
	NEX length (mm)	−0.599	0.159	−0.579	−0.926	-	−0.273		0.001			
2	(Constant)	27.106	129.611		−237.978	-	292.190		0.836		−0.033	
	Height (cm)	0.173	0.795	0.040	−1.452	-	1.799	0.829				

NEX: Nose-Ear-Xiphoid; β: regression coefficient; Std β: standardized regression coefficient; Adj R^2^: adjusted R^2^.

## Discussion

This study revealed the tremendous variability of NG tube placement while using the NEX body surface method to estimate the inserting depth. We found that 96.7% of the patients (29 in 30 patients) failed to have the NG tube placed correctly, and 46.7% patients (14 in 30 patients) had one or more side-holes in both the esophagus and the stomach area. Fifteen patients (50.0%) had inappropriate NG tube depth and could not even pass through any side-holes across the gastroesophageal junction. Body height had the greatest statistical correlation with NG tube length, as compared with the NEX, GX and GU body surface methods.

Previous studies used gastric endoscopes and even laparotomy to check the length of NG tubes. [15] Some others identified the fluorescence markings on plane films. [16] But these methods were either invasive with unpredictable risks or harmful due to radiation exposure. Besides, vague shadows and stomach confines may lead to misinterpretation of tube-tip locations on plane film radiographs, especially when the alignment of the NG tube parallel to radial beams. In the current study, the insertion depths of NG tubes were measured precisely by multiplanar reconstruction algorithm of PET-CT. This is a new technique which can discover the correct catheter length inside the human body in 3-dimensional space.

The traditional NEX method to determine the depth of gastric tube placement has been used for a long time [5], [10], but the prior literatures have not thoroughly discussed the properties of this estimation method except in a child population. In 1992, Scalzo reported a 50% (7/14) malposition rate in pediatric patients. [17] In adults, previous studies focused on the confirmation tests of proper location to prevent displacement in the trachea or other areas. Auscultating borborygmus over the epigastrium during air injection, aspirating gastric contents with measurement of the pH [18], pepsin [19], bilirubin, and trypsin levels [20], examining aspirate characteristics [21], and detecting the carbon dioxide level at the proximal end of the tube of have been reported. [12] But these confirmation tests might provide incorrect data or only partial data about side-hole placement into the stomach.

Correct placement of these tubes provides proper functionality and maximizes benefits and minimizes risks. It is recommended that caregivers should verify the placement of NG tube at initial use, before a feeding, before administration of a medication, and every shift unless otherwise indicated. [9] But there are still many aspirations that predispose tube-fed patients to pneumonia in clinical practice, especially those who are facing consciousness disturbance and those that are critically ill. Langmore SE prospectively enrolled 189 patients and determined that tube feeding was an independent predictor of aspiration pneumonia. [11] Another cross-sectional study included 102,842 patients showing that tube feeding is the third strongest of eighteen significant predictors of aspiration pneumonia. [22] Consequently, we assumed that the side-hole displacement may contribute to these complications.

In our study, only one patient successfully placed all of the side-holes into the stomach according to the NEX body surface estimate method and 29 people (96.7%) failed. This emphasizes that the NEX body surface method is unreliable to predict the ideal insertion depth of the NG tube and usually results in underestimations. The only patient with correct placement of complete side-holes may have had the most effective NG tube function and the lowest regurgitation or aspiration risk. Fifteen people (50%) failed to pass through any side-holes and confronted some obstacles while undergoing the physician tests with regards to the function of inserting tube. However, these problems will remind the practitioner of unsuccessful placements; the patients usually receive reintubation with deeper inserting length. But in cases where the NEX estimate method fails, there is no advised additional insertion length in the related literatures. [5] With the current study design, at least 95 mm should be inserted forward to reach a correct and safe placement. Gradual push of tube with every small distance might increase the risk of partial placement of side-holes. However, these were usually seen in clinical practice.

There are 14 patients with one or more side-holes in both the esophagus and the stomach, and we assume that these patients may struggle with the highest risk of aspiration of tube or gastric content. Human esophagi are usually flaccid with nearly no air content; the gastric substance still could be drained from the distal side-holes. Examination of suction materials may be masked with the inflow. And likewise, air injection to NG tube and leak through the distal side-hole also results in a false negative finding of auscultating borborygmus test. [23] In these situations, previous certification tests could not help to evaluate the placement correctly. The misunderstanding of incorrect placement and consequently pouring of feeding substance or medication may lead to many complications, such as mucosa inflammation and esophagitis. [24] Metheny NA reported increased risk of pulmonary aspiration if the proximal end of the NG tube extended only to the esophagus. [13] It might be that the leakage from side-holes above the gastroesophageal junction could be prone to regurgitation and gagging, especially for patients in a supine position. Previous literature showed that an elevated head-of-bed position is helpful in reducing aspiration and pneumonia. [25]-[26] And it is crucial that these patients are in an upright posture and use gravity to pass contents into stomach. Besides, the stretch receptors in the esophageal lining may be stimulated by leakage from side-holes in the esophagus. A local reflex response causes a secondary peristaltic wave around the bolus, forcing it further down the esophagus. [27] It is maybe the reason why tube content goes through the gastrointestinal tract by using the traditional NEX method with only partial side-hole placement. And we can speculate that the underinsertions are causing many complications that are rarely mentioned.

Three body surface methods which include NEX, GX, and GU measurement were tested in the current study. In linear regression models, height is the most valuable predictor of NG length. The correlation coefficient is 0.691 and the adjusted R^2^ is 0.459. Literaturally, there are some direct morphological measures to estimate internal orogastric or nasogastric distances in child. Klasner AE reported a new gastric formula which was based on the height, and the tube insertion depth was better than NEX method in a pediatric population. [16] For better accuracy, increased consistency, and decreased variability of NG tube placement, further body surface methods should be tested and compared with the estimate method by body height.

Comparisons between the 15 patients who failed to pass through any side-hole with the other 15 patients showed that they had significantly shorter EX distances. (mean = 348.1 vs. 368.9 mm, p = 0.02) But there was no difference between the NE distances. (mean = 151.9 vs. 154.0 mm, p = 0.77) Interestingly, our result suggest that the EX distance may determine the success rate of proper distal tube placement. And people with shorter EX had the higher failure rate. In linear regression models, there were strong negative correlations between the NEX length and the additional lengths to secure side-holes placement. (Std β = −0.579, p = 0.001) The longer NEX length measured, the less additional distance should be added. However, height did not show statistical association with the additional length to secure side-holes placement. We cannot use patient height as the only variable to determine the plus depth. It may be that these body surface methods were unreliable in people with smaller NEX lengths, such as people with short necks or small thoracic cages. Medical administrator should pay more attention to these patient groups, especially when using body surface NEX method to interpret the NG tube inserting length.

### Limitations

This study has an apparent limitation of sex distribution deviation, as there were 27 men and 3 women that finally participated. This is because of the retrospective study design and use of PET-CT scan to measure NG tube inserting length. Most of these patients had the diagnosis of oral or nasopharyngeal cancer. Such diseases are more common in men, and with the male-female ratio of approximately 3∶1 for oral cavity and pharyngeal cancers. [28]

Another limitation with regards to the participants was related to race. All 30 patients are Han Chinese race and might have relatively shorter stature, bony structure, or less prominent facial features. This difference may influence the study results. Third, small number of participants limited the results with regards to safety and correlation issue of NEX method. It's inappropriate to conclude advised further insertion depth of NG tube while using NEX method in current study. Further trials which include balanced gender, various races and a greater number of patients are warranted.

There is clinical significance in the daily and multi-day variation of the position of the distal NG tube, particularly after transport and patients care. The PET-CT scan with reconstruction algorithm has a good ability to investigate the precise location of NG tip, but this retrospective study did not repeat the evaluation. We suggest further studies could focus more on this valuable situation.

There are different types and brands of NG tubes modified for feeding, perioperative acute phase, traumatic or poisoning use. One kind of commercial NG tubes was used in current study which had four side-holes and the distance between catheter-tip was 95 mm. The last limitation is that the study result may change while using different kinds of NG tubes. And it differed especially from the varied length between the proximal side-hole and the catheter-tip.

## Conclusion

It can be concluded that the traditional NEX body surface method of determining depth of gastric tube insertion is inappropriate in adult patients and is inconsistent with ideal NG tube length. We recommend that the length of inserting tubes should not be determined by the NEX method even when the borborygmus auscultation or aspirating gastric contents tests show normal findings.

Further prospective, large number and well-designed studies were warranted to identify more reliable, invariable and convenient body surface methods. Thus, these might improve the functionality and feasibility of NG tubes and prevent complications.
