# Correction: Potential Risk of Malposition of Nasogastric Tube Using Nose-Ear-Xiphoid Measurement

**DOI:** 10.1371/journal.pone.0096438

**Published:** 2014-04-23

**Authors:** The PLOS ONE Staff

The affiliations of the last author Ta-Sen Wei are incorrect. The correct affiliations are: (1): Department of Physical Medicine and Rehabilitation, Changhua Christian Hospital, Changhua, Taiwan and (4): School of Medicine, Chung Shan Medical University, Taichung, Taiwan.
